# Supplementary material for: Unveiling the circRNA-Mediated Immune Responses of Western Honey Bee Larvae to Ascosphaera apis Invasion
Source: Int J Mol Sci. 2022 Dec 29;24(1):613. doi: 10.3390/ijms24010613 (PMC9820429; doi:10.3390/ijms24010613)
Supplement: Supplementary file 1 [file ijms-24-00613-s001.zip › ijms-2084491-supplementary.pdf]

Table S1 Summary of mapping of anchors reads to the reference genome of *A. mellifera*

sample	anchors reads	anchors reads mapped in reference genome	map rate (%)
AmCK1	7 471 892	3 160 593	42.30
AmCK2	6 905 870	3 734 903	54.08
AmCK3	8 328 540	4 154 993	49.89
AmT1	7 893 288	3 862 442	48.93
AmT2	7 350 996	3 826 332	52.05
AmT3	5 845 700	3 040 217	52.01

Table S2 Summary of target mRNAs involved in immune pathways

Pathways	AmCK1 vs AmT1 comparison group	AmCK2 vs AmT2 comparison group	AmCK3 vs AmT3 comparison group
Lysosome	1	2	0
Endocytosis	1	0	1
Phagosome	0	1	1
Autophagy	0	0	1
Apoptosis	0	0	2
MAPK signal pathway	2	2	1
Jak-STAT signal pathway	0	1	1
Toll and Imd signal pathway	0	0	1

Table S3 Detailed information of primers used in this work

Name	Sequence (5′-3′)
novel_circ_000007-F	CGAGGTCAAACTGTGTCCCGTA
novel_circ_000007-R	TCCACCAGGTCCAACCGAACT
novel_circ_001116-F	AATTGACCGATGCTTACGAGGC
novel_circ_001116-R	TTTGTTCCCGCCCACTTTGC
novel_circ_001620-F	ACACTCCAGCTGGGTAAAGCTAGATTA
novel_circ_001620-R	TTTGTTCCCGCCCACTTTGC
novel_circ_000465-F	CCATCAACACGGGAACCACA
novel_circ_000465-R	GGCTGAACTTCCCTCGTGTC
novel_circ_000474-F	GCATAGTCAGCACCACCAGT
novel_circ_000474-R	GCTGCCTTGGCTGGATGATA
novel_circ_001617-F	GAAATCGCGGTCACATTGGC

novel_circ_001617-R	CGTCAAAGTGAATGCAGTGGG
novel_circ_000573-F	AGGATTCGCCGCTCGTCATTG
novel_circ_000573-R	GTGCCATCTCGCTGGACATCTC
novel_circ_000264-F	GCGAAGCAAAGGACGACGAGTC
novel_circ_000264-R	TGGAAGGAACGATGGTGAGGATGA
novel_circ_000653-F	AGACGTGACCGACTCGAAGGA
novel_circ_000653-R	GAACGGCAGACGGTGATCCAA
novel_circ_000400-F	CCTGGTACTCGTGTTCTCCG
novel_circ_000400-R	CCTCGATCTTCCCACCATCG
novel_circ_001048-F	CTCGAGGATGATGTCATTGATCT
novel_circ_001048-R	GGGAGCTTACCATCCTTCGT
novel_circ_001377-F	GGACGAAGAGACAGTTCCACA
novel_circ_001377-R	GGTGGCTCAGGATCGGTAGA
novel_circ_000408-F	AGATTCAGCCAGCATCGTCACA
novel_circ_000408-R	TCCTCCTCCTCCTCCTCCTGTT
novel_circ_000465-F	CCATCAACACGGGAACCACA
novel_circ_000465-R	GGCTGAACTTCCCTCGTGTC
novel_circ_000573-F	AGGATTCGCCGCTCGTCATTG
novel_circ_000573-R	GTGCCATCTCGCTGGACATCTC
novel_circ_000846-F	AAGACTTCCGCTTCCGAACCAA
novel_circ_000846-R	TCTCTTTCCAACTCGCCGCTAA
novel_circ_000922-F	CGGCCATCAGATTCAGCAGCA
novel_circ_000922-R	TGCGTGCATTGCAGCTTCAC
novel_circ_001116-F	AATTGACCGATGCTTACGAGGC
novel_circ_001116-R	TTTGTTCCCGCCCACTTTGC
novel_circ_001374-F	AACGAGGACAAGGAAGAT
novel_circ_001374-R	ATTGATCCGACAGTGGTT
novel_circ_001556-F	CGATGTCGTTGGAGCAGGAAGT
novel_circ_001556-R	GCGGACGTTGGTGTCGTTGAA

novel_circ_001620-F	TCAGACAAGAGCCGCAGCATTC
novel_circ_001620-R	CGGACGACGGTGAGAGATGGAA
novel_circ_000774-F	AGAATGTCGACTTGCACCGA
novel_circ_000774-R	GTGTGGGAAGAGGAGCAGTC
novel_circ_000344-F	CGCCCAGTCCAGTTATCGTT
novel_circ_000344-R	GTGGCTCCGAGATCAACACA
novel_circ_001194-F	TATGAGCACCAGCAGCTACG
novel_circ_001194-R	AGGGACTTCCTCTACCGCAT
actin-F	CACTCCTGCTATGTATGTCGC
actin-R	GGCAAAGCGTATCCTTCA
